# N-myc downstream-regulated gene 1 promotes apoptosis in colorectal cancer via up-regulating death receptor 4

**DOI:** 10.18632/oncotarget.19658

**Published:** 2017-07-28

**Authors:** Xian Zhang, Bo Feng, Fan Zhu, Chaoran Yu, Jiaoyang Lu, Meng Pan, Zirui He, Xiongzhi Wangpu, Jing Sun, Xiao Yang

**Affiliations:** ^1^ Shanghai Institute of Digestive Surgery, Ruijin Hospital, Shanghai Jiao Tong University School of Medicine, Shanghai 200025, China; ^2^ Department of General Surgery, Division of Gastrointestinal and Colorectal Surgery, Shanghai East Hospital, Tongji University, Shanghai 200120, China; ^3^ Department of General Surgery, Ruijin Hospital, Shanghai Jiao Tong University School of Medicine, Shanghai 200025, China

**Keywords:** apoptosis, colorectal cancer, death receptor, NDRG1, TRAIL

## Abstract

The aim of this study was to evaluate the clinical significance of N-myc downstream-regulated gene 1 (NDRG1) in colorectal cancer (CRC) patients and to explore the mechanisms governing the role of NDRG1 in apoptosis of CRC cells. In the current study, we found that NDRG1 was a prognostic marker of CRC patients. Moreover, NDRG1 expression negatively correlated to tumor size and clinical TNM stage, suggesting that NDRG1 might act as a tumor suppressor by inhibiting proliferation or inducing apoptosis in CRC. Consistently, substantial apoptosis was observed *in vitro* and *in vivo* in the presence of NDRG1. From a mechanistic standpoint, we discovered that NDRG1 was able to prevent death receptor 4 from degradation induced by MARCH-8, a member of the membrane-associated RING-CH (MARCH) ubiquitin ligase family. As a consequence, CRC cells expressing NDRG1 were more sensitive to reagents targeting death receptors such as tumor necrosis factor-related apoptosis-inducing ligands (TRAIL). Additionally, the pro-apoptotic effect of NDRG1 was also validated in mouse xenograft model. In conclusion, our results provided further insights of the pivotal role of NDRG1 in apoptosis initiated by death receptors and demonstrated a novel marker to predict the sensitivity of CRC to TRAIL treatment in future clinical study.

## INTRODUCTION

Colorectal cancer (CRC) is one of the most prevalent alimentary tract tumors leading to the third cancer-related death across the world [1]. More than 1.2 million patients are diagnosed with CRC every year [2]. Dramatic progress has been achieved in surgery, neoadjuvant therapy, and adjuvant chemotherapy [3]. Despite these improvements, tumor progression is still the major cause of cancer-related death [4]. So, clearly, new approaches are urgently needed for further modification of treatment strategy of CRC. The identification of molecules regulating cancer cell death in response to chemotherapy is of great importance, as such molecules may constitute important clinical predictive markers of response to chemotherapy or novel therapeutic targets [5].

One tumor suppressor that has recently attracted increasing attention is NDRG1. This molecule was identified as a metastasis suppressor gene in human breast [6, 7], prostate [8] and colorectal cancers [9], in which it was found to reduce cell growth and suppress metastasis both *in vitro* and *in vivo*. Moreover, NDRG1 is also reported to have effect on genome stability [10], apoptosis [11], even cancer cell stemness [12]. Despite the ubiquitous link of NDRG1 to various cellular events, however, mechanisms of these actions are yet to be fully elucidated, especially how NDRG1 exerts its special function in apoptosis.

Tumor necrosis factor-related apoptosis-inducing ligands (TRAIL) is a member of the tumor necrosis factor (TNF) family that can selectively trigger rapid apoptosis in various tumor cells while leaving most of the normal cells unharmed [13, 14]. Thus, there is growing interest in considering TRAIL as a therapeutic strategy, as TRAIL has been shown to exert substantial antitumor effects without resulting in serious side effects in several xenograft model [15]. Moreover, recombinant TRAIL and agonistic antibodies that trigger death receptor activity are under clinical evaluation in cancer patients [16–18]. Although many tumors are sensitive to TRAIL-mediated apoptosis, the majority, including CRC, remains resistant [19]. This resistance is conferred by a number of molecular changes including the down-regulation of death receptors [13]. These findings led us to hypothesize that the anti-tumor activity of NDRG1 might lie in its ability to modulate death receptors and enhance the cytotoxic effect of TRAIL on CRC. Thus, the present study aimed to explore the role of NDRG1 in TRAIL-induced apoptosis and to investigate of potential mechanisms of this action.

## RESULTS

### NDRG1 was a prognostic marker for CRC patients

**Figure 1:**
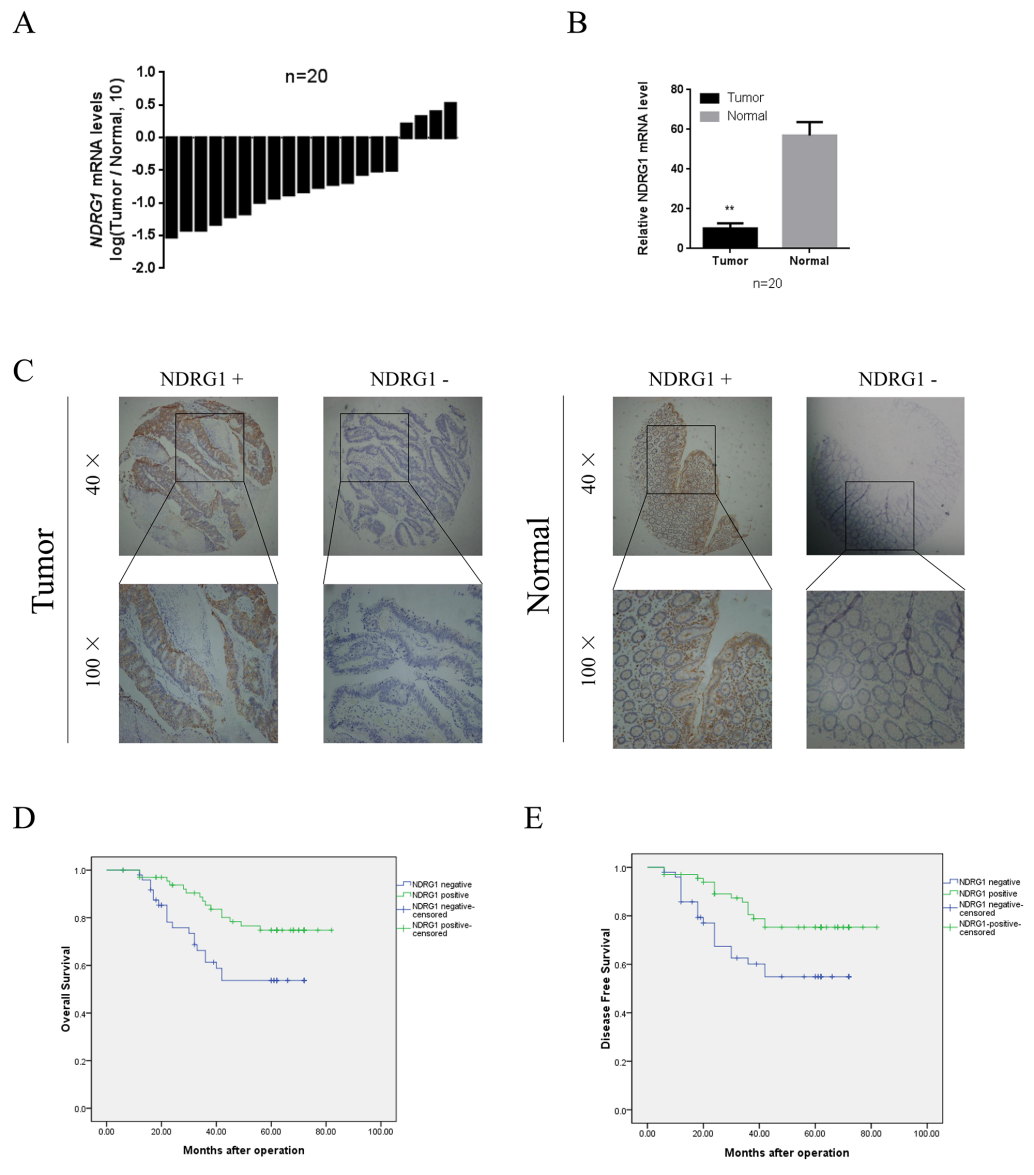
Expression and clinical significance NDRG1 in CRC patients **(A)** qRT-PCR analysis showing NDRG1 expression in 20 paired CRC samples that were randomly selected from the 116 CRC cases, by random numbers generated with SAS software. **(B)** Quantative analysis of qRT-PCR showing that significant decrease of NDRG1 expression in tumor tissue as compared to paired normal tissue. **(C)** NDRG1 expression levels in tumor tissues and the paired normal tissues were evaluated by immunohistochemical staining with tissue microarray. **(D)** CRC patients with positive expression of NDRG1 presented longer overall survival and disease free survival **(E)** compared with those of negative expression.

To determine the role of NDRG1 in human colorectal cancer development, we first evaluated mRNA expression levels in 20 pairs of cancer specimens randomly selected from the cohort. We compared the endogenous NDRG1 expression in human CRC with that in adjacent normal tissue by quantitative RT-PCR. As shown, NDRG1 mRNA levels were significantly decreased in tumor tissues relative to matched normal tissues (Figure 1A and 1B). Moreover, tissue microarray containing 116 pairs of cancerous and matched normal tissue was analyzed by immunohistochemical staining (Figure 1C). Among these cases, positive NDRG1 expression was observed in 59.5% (69/116) of normal tissues. Meanwhile, in the paired CRC tissue, only 30.2% (35/116) cases showed positive signal (*P* < 0.01, Table 1).

**Table 1:** Relationship between NDRG1 expression and clinical-pathologic features in 116 CRC patients

Variable	Case	NDRG1 expression		*P* value
		Positive	Negative	
Tissue				0.000
Normal	116	69	47	
Cancer	116	35	81	
Gender				0.533
Male	68	19	49	
Female	48	16	32	
Age				0.967
≤ 65	56	17	39	
> 65	60	18	42	
Location				0.251
Left hemicolon	16	5	11	
Right hemicolon	33	10	23	
Sigmoid colon	22	3	19	
Rectum	45	17	28	
Tumor size				0.004
≤ 4 ×3	49	22	27	
> 4 ×3	67	13	54	
Local invasion				0.042
T1 + T2	52	21	31	
T3 + T4	64	14	50	
Lymphatic invasion				0.014
N0	46	20	26	
N1+N2	70	15	55	
Remote metastasis				0.150
M0	99	27	72	
M1	17	8	9	
TNM stage				0.014
I + II	46	20	26	
III + IV	70	15	55	

To further investigate the clinical significance of NDRG1 in CRC, we then performed a correlation analysis to assess the relationship between NDRG1 expression and clinical variables by dividing patients into two groups: NDRG1 negative or NDRG1 positive. Our data showed a negative correlation between NDRG1 expression and tumor size, local invasion, lymphatic invasion, and TNM stage (Table 1). Kaplan–Meier analysis revealed that patients in the NDRG1 negative expression group had a significantly poorer 5-year overall survival (OS) than those in the NDRG1 positive group (log-rank = 6.507, *P* = 0.011, Figure 1D). Additionally, NDRG1 positive expression was associated with a longer disease-free survival (DFS) time (log-rank = 6.259, *P* = 0.012, Figure 1E). These data suggested that NDRG1 was down-regulated in CRC and could function as a prognostic marker for both OS and DFS in CRC patients.

### NDRG1 suppressed cell viability by inducing apoptosis in CRC cells

**Figure 2:**
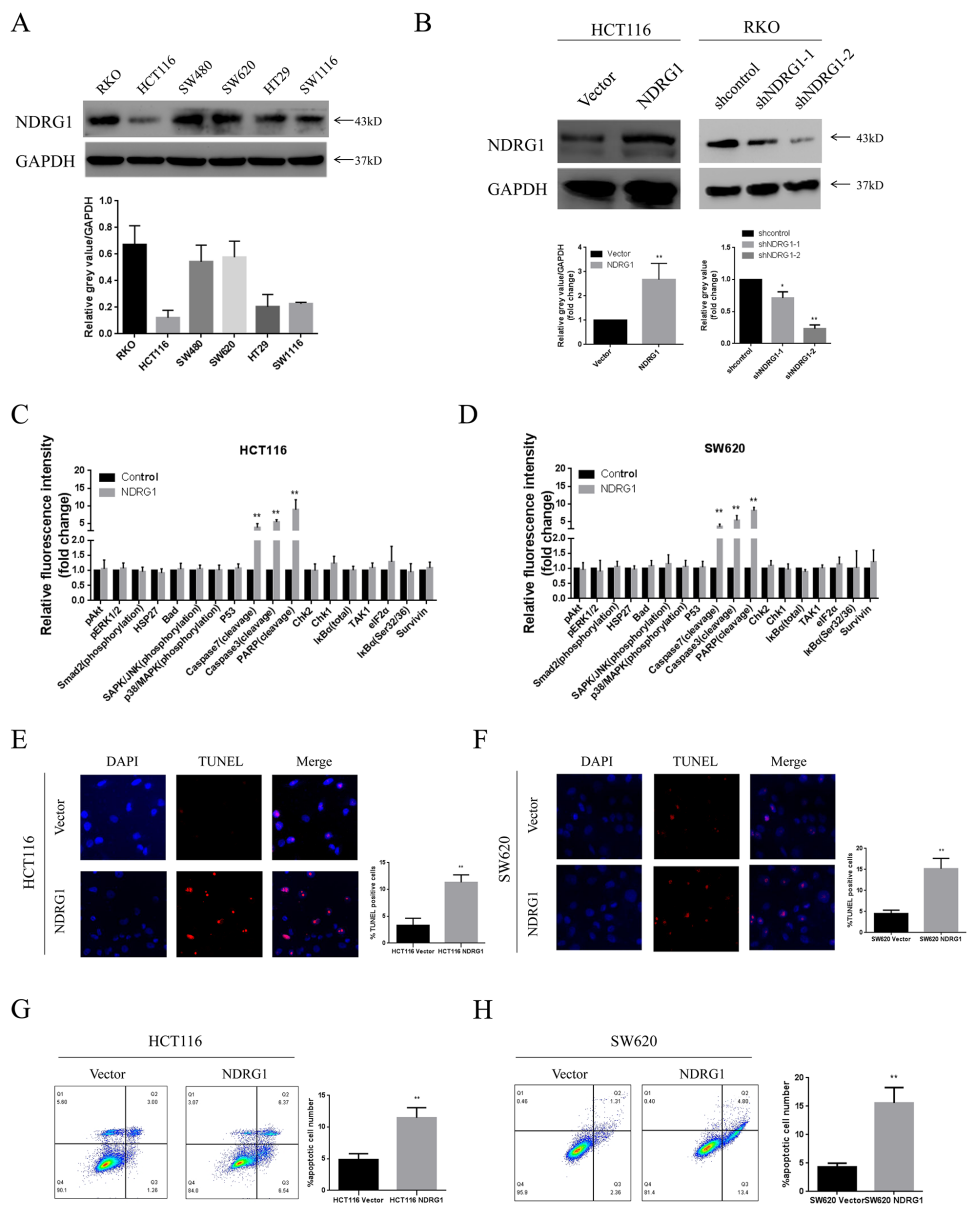
NDRG1 suppressed CRC cell viability by inducing apoptosis **(A)** Expression of NDRG1 in colorectal cancer cell lines. Endogenous protein levels were determined by western blot assay. GAPDH was used as a loading control. **(B)** Over-expression and knockdown of NDRG1 were performed in HCT116 and RKO cells. The more effective shRNA was chosen for further research. **(C)** Pathscan array showing elevated cleavage of caspase-7, caspase-3 and PARP after NDRG1 over-expression in HCT116 and SW620 **(D)** cells. **(E)** Prominent increase of positive signal of TUNEL staining was observed in NDRG1 over-expressed models of HCT116 and SW620 **(F)** cells. Magnification, ×200. **(G)** Larger apoptotic cell population was detected by flow cytometry assay in NDRG1 over-expressed HCT116 and SW620 **(H)** cells as compared to control cells. Data was representative of three independent experiments, bars indicated S.D., *, *P* < 0.05, **, *P* < 0.01.

Having demonstrated that NDRG1 was inversely associated with tumor size in clinical samples, we then aimed to explore the underlying mechanisms. We first established NDRG1 over-expressed and knockdown cell models in CRC cell lines. The expression levels of NDRG1 in six CRC cell lines were analyzed with western blot assay. As shown, NDRG1 was differently expressed among these cell lines and especially high in RKO cells, but low in HCT116. Other cell lines including SW480, SW620, HT29 and SW1116 showed moderate levels of NDRG1 (Figure 2A). HCT116 was selected to establish NDRG1 over-expression cell model. In the mean time, two hairpin sequences were designed to knock down NDRG1 in RKO cells. ShNDRG1-2 was chosen for further research. The effect of over-expression or knockdown was confirmed by western blot (Figure 2B). Subsequently, potential alterations of several pathways relevant to cell proliferation or apoptosis were analyzed with a pathscan array in NDRG1 over-expressed cells. As shown in Figure 2C and 2D, elevated cleavage of caspase-7, caspase-3 and PARP were revealed after NDRG1 over-expression. In contrast, no significant difference were detected in proliferation pathways, which suggested that the decreased tumor sizes in NDRG1-positive patients were attributed to enhanced apoptosis instead of blocked cell proliferation. Consistently, significantly positive signal of TUNEL staining was seen in NDRG1 cells of HCT116 and SW620 cells (Figure 2E and 2F). Moreover, Annexin V/PI assays showed a dramatic increase of apoptosis rate in NDRG1 cells as compared to control cells (Figure 2G and 2H). These results indicated that NDRG1 suppressed cell viability via amplifying the magnitude of apoptosis in CRC cells.

### NDRG1 induced apoptosis via up-regulating death receptor 4

**Figure 3:**
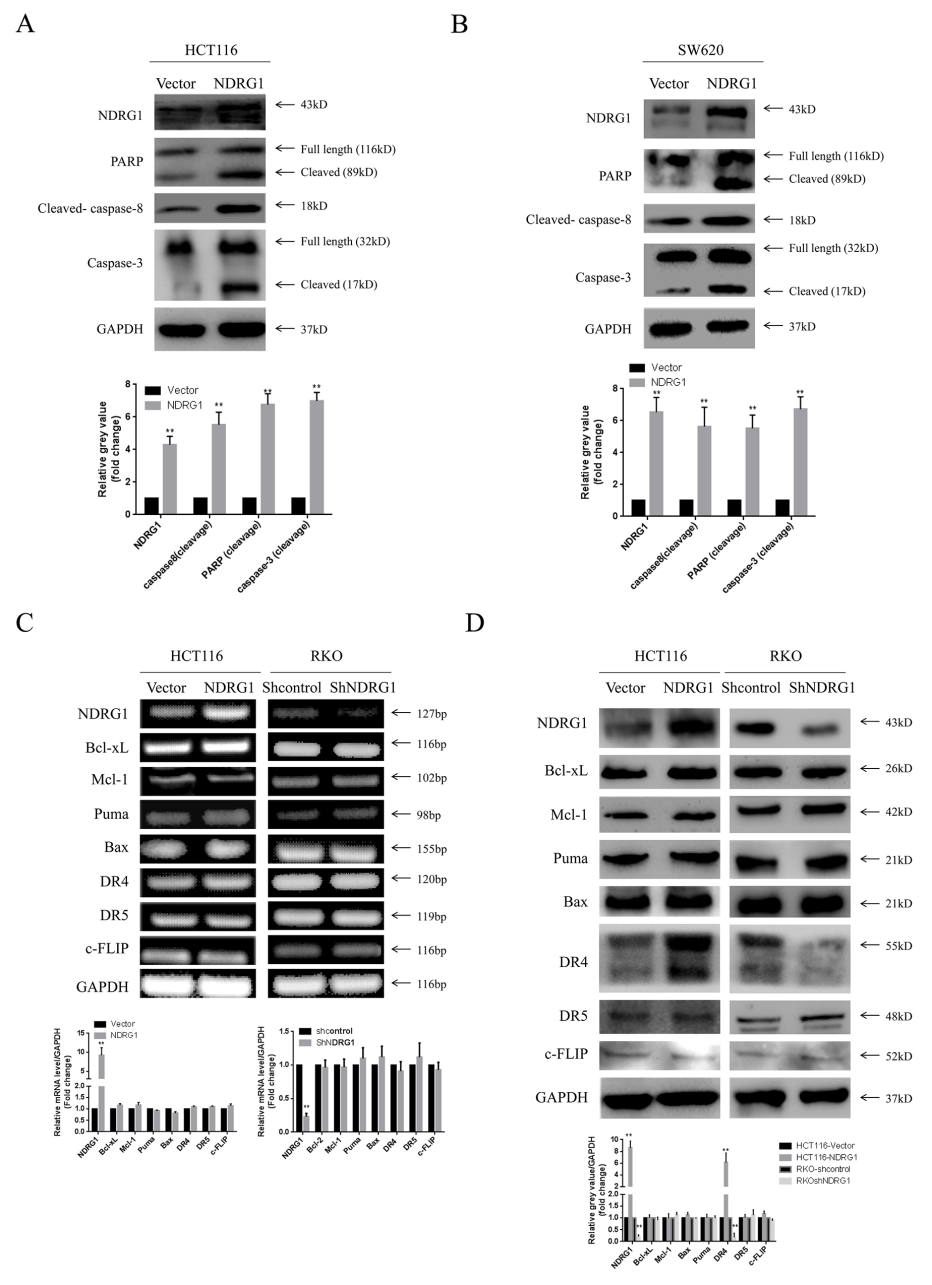
NDRG1 induced apoptosis via activating death receptor pathway **(A)** NDRG1 ectopic expression led to cleavage of caspase-8, casepase-3 and PARP in HCT116 and SW620 **(B)** cells. **(C)** RT-PCR showing no alterations in mRNA level several main regulating factors of apoptosis. **(D)** Among these molecules, protein level of death receptor 4 was increased upon NDRG1 expression. Data was representative of three independent experiments, bars indicated S.D., *, *P* < 0.05, **, *P* < 0.01.

We next sought to investigate the pathway by which NDRG1 triggered apoptosis in CRC. Caspase-3 and casepase-7 are members of the caspase (cysteine aspartate protease) family, and have been shown to be executioner proteins of apoptosis [20]. They exist as inactive proenzymes that undergo proteolytic processing induced by upstream caspases (caspase-8, -10) upon cell death stimuli. Given the fact that NDRG1 might trigger apoptosis in caspase-3 and -7 dependent manners (Figure 2C and 2D), western blot assay was performed to analyze levels of caspase-8. As expected, the active form of caspase-8 was elevated upon NDRG1 expressing (Figure 3A and 3B).

**Figure 4:**
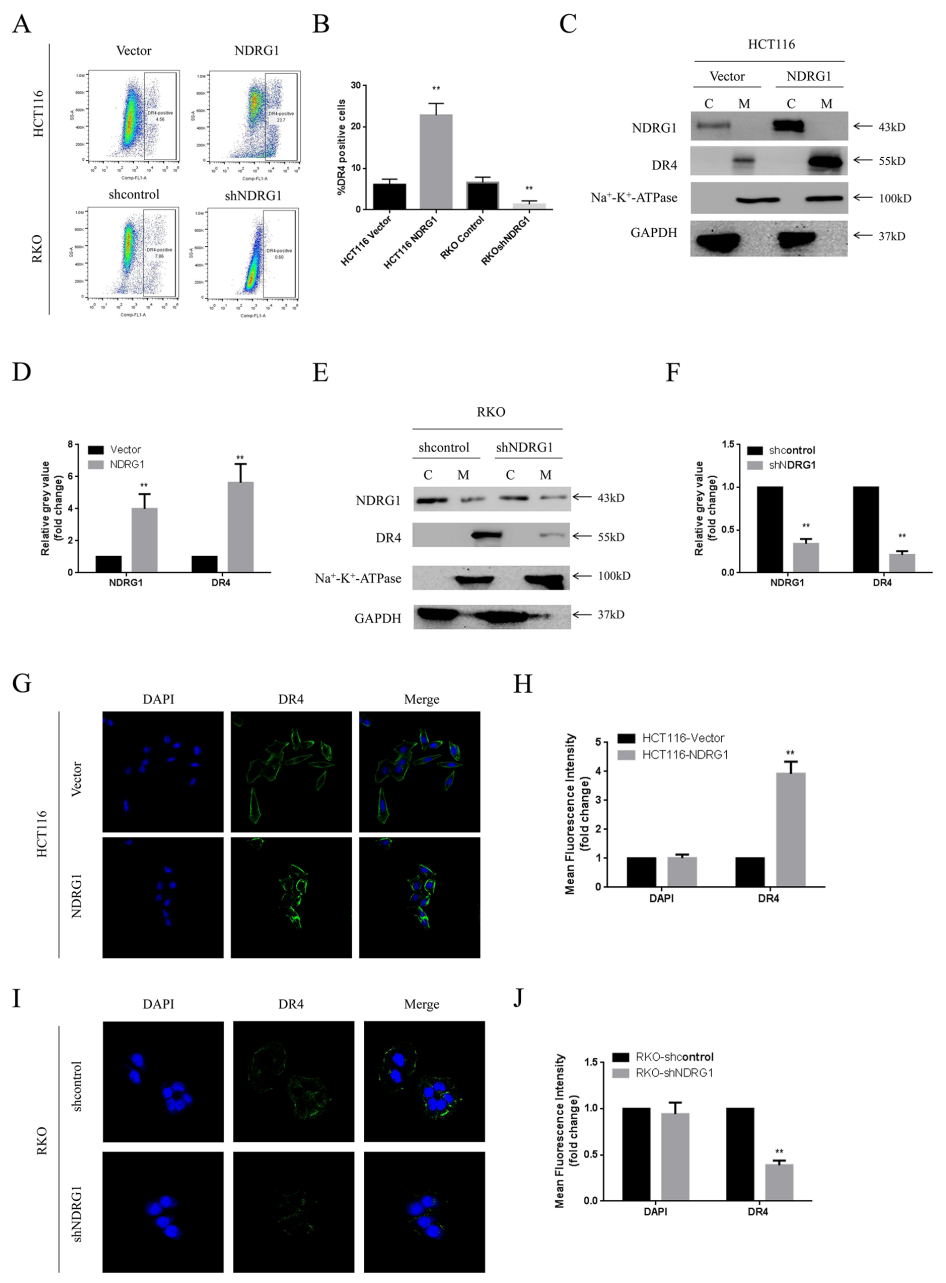
NDRG1 up-regulated death receptor 4 protein level **(A)** Levels of DR4 were analyzed with flow cytometry assay. **(B)** Quantitative analysis of DR4 levels in NDRG1 derivates of HCT116 and RKO cells. **(C, D)** Fractions of NDRG1 and control cells were analyzed with western blots showing that elevation of DR4 in membrane of NDRG1 cells. **(E, F)** Western blots showed that depletion of NDRG1 resulted in decrease of DR4 in cell membrane. **(G, H)** Immunofluorescent staining of death receptor 4 in HCT116-NDRG1 and vector control cells. Green: DR4; blue: nuclear (DAPI) staining; magnification, ×200. **(I, J)** Immunofluorescent staining of death receptor 4 and quantification of mean fluorescence intensity in RKO-shNDRG1 and control cells. Data was from three independent experiments, bars indicated S.D., *, *P* < 0.05, **, *P* < 0.01.

To gain more insight into the apoptotic signaling triggered by NDRG1, we tried to test the main regulating factors of death receptor signal pathway and mitochondrial pathway by RT-PCR and western blot assay. However, no difference in mRNA levels was detected in NDRG1 over-expressed or knockdown cells (Figure 3C), suggesting transcriptional mechanism was not involved in this process. Nevertheless, in western blot assay, death receptor 4 (DR4) was found to accumulate in NDRG1 cells and to decrease in shNDRG1 cells (Figure 3D). To further verify this, flow cytometry assay was performed to analyze DR4-positive rates in NDRG1 over-expressed and knockdown cell models. As shown, significant positive signal of DR4 was detected in HCT116-NDRG1 cells while RKO-shNDRG1 cells showed negligible signal (Figure 4A and 4B). Moreover, the above studies were complemented using cellular fractionation and immunofluorescence to assess the effect of NDRG1 on the levels of DR4 in CRC cells. As shown, over-expression of NDRG1 resulted in a marked increase of DR4 protein in the membrane fractions (Figure 4C and 4D) whereas knockdown of NDRG1 led to the opposite (Figure 4E and 4F). In agreement with this, immunofluorescence staining of DR4 showed a significant increase or decrease in fluorescence intensity in the NDRG1 over-expression (Figure 4G and 4H) and knockdown (Figure 4I and 4J) cells, respectively. To provide more solid evidence, we performed selective knockdown of DR4 in HCT116-vector and -NDRG1 cells and measured the effect of this action on apoptosis. As shown, the increased apoptosis induced by NDRG1 expression was almost abrogated by loss of DR4 (Supplementary Figure 1A). Taken together, these data suggested that the promoting effects of NDRG1 on apoptosis in CRC cells were attribute to its ability of up-regulating death receptor 4.

### NDRG1 suppressed the ubiquitination of death receptor 4

**Figure 5:**
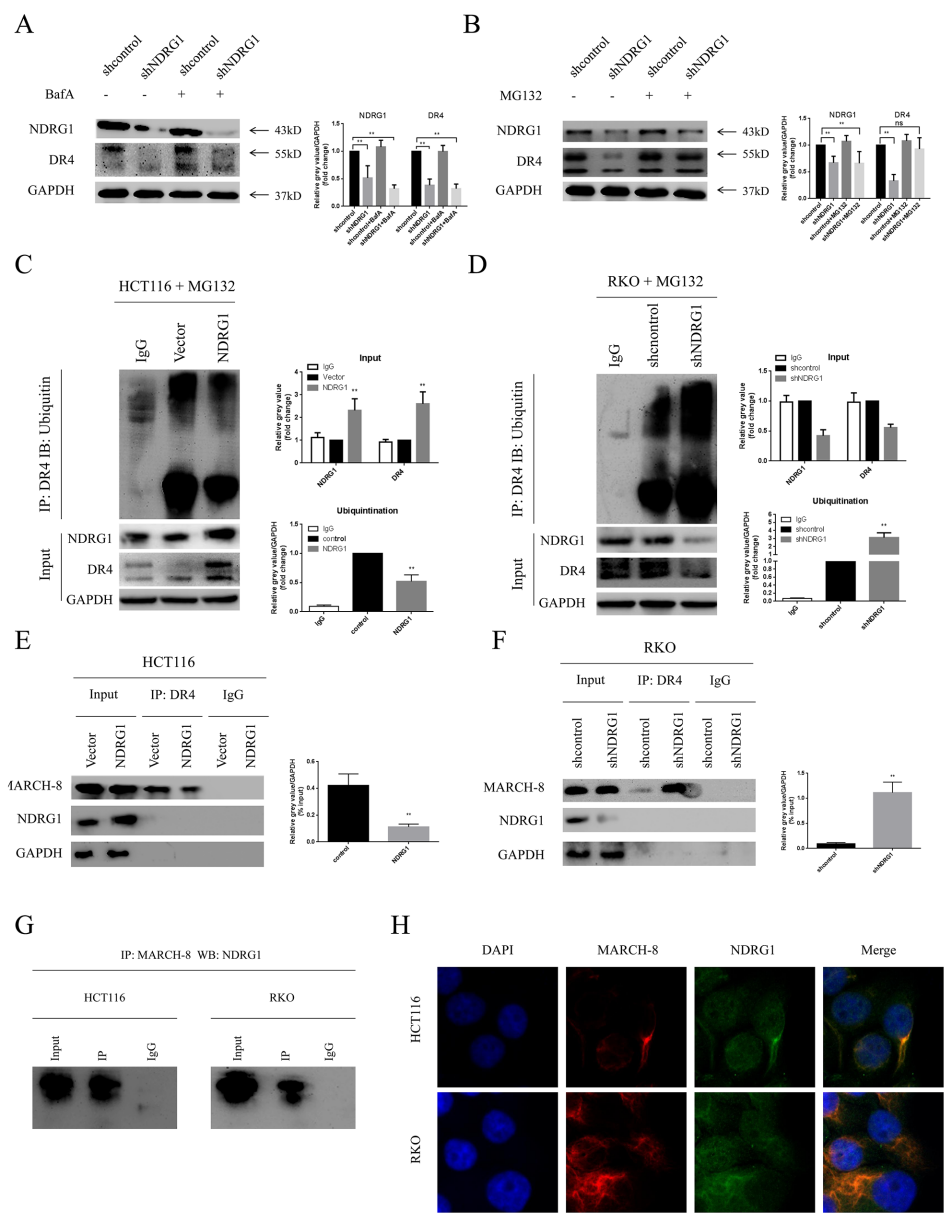
NDRG1 suppressed ubiquitination of death receptor 4 **(A)** Effect of BafA on protein levels of death receptor 4 was measured with western blots. **(B)** MG132 restored protein levels of DR4 in shNDRG1 cells. **(C)** Ubiquitination levels of DR4 were analyzed with western blots in NDRG1 over-expressed and knockdown **(D)** cells. **(E)** Interaction of DR4 and MARCH-8 was analyzed with co-IP assay in NDRG1 cells and shNDRG1 **(F)** cells. **(G)** co-IP assay showing NDRG1 directly binded with MARCH-8. **(H)** Immunofluorescent staining showed co-location of NDRG1 and MARCH-8. Green: NDRG1; red: MARCH-8; blue: nuclear (DAPI) staining; magnification, ×200. Data was shown as mean ± S.D. of triplicate experiments. *, *P* < 0.05, **, *P* < 0.01.

To figure out how NDRG1 modulate DR4, HCT116-NDRG1 cells were treated with cycloheximide (CHX, 20μg/ml) for a period of 5 hours. Then the protein level of DR4 was analyzed with western blots. As protein synthesis was blocked, the level of DR4 was decreased relative to untreated cells. However, NDRG1 cells still showed a significant accumulation of DR4 comparing to vector control (Supplementary Figure 1B). This result suggested that NDRG1 up-regulated protein level of DR4 by preventing it from degradation instead of promoting its synthesis. Cytoplasmic and membrane proteins are often degraded in either lysosome or proteasome. Therefore, inhibitors for both pathways were used to explore how DR4 degrade in CRC cells. Our results showed that the lysosome inhibitor BafilomycinA1 (BafA) had no effect on DR4 degradation in shNDRG1 cells (Figure 5A). In contrast, MG132, a proteasome inhibitor, markedly restored the decreased DR4 protein level induced by NDRG1 depletion (Figure 5B), indicating DR4 mainly degraded via proteasomal pathway. Proteins degraded by proteasome system are most often conjugated to multiple copies of the small protein ubiquitin through covalent bonds, resulting in the formation of ubiquitinated forms of proteins. To assess the potential effect of NDRG1 on the ubiquitination of DR4, immunoprecipitation assay was performed in both NDRG1 over-expressed and knockdown cells. As expected, DR4 underwent remarkable poly-ubiquitination after depletion of NDRG1 (Figure 5D), whereas NDRG1 over-expression prevented it from being ubiquitinated (Figure 5C).

A recently discovered group of ubiquitin ligases that targets membrane molecules is the membrane-associated RING-CH (MARCH) family. Upon exogenous expression, a number of these ligases can affect the trafficking of membrane molecules. Previous studies reported that MARCH-8 could attenuate DR4 cell surface expression and apoptosis signaling by virtue of its ligase activity [21]. MARCH-8 interacted with DR4 and subsequently mediated the conjugation of DR4 and ubiquitin. As MARCH-8 is the only reported E3 ligase responsible for the stability of DR4 [21], NDRG1 may modulate the ubiquitination levels of DR4 via affecting the levels of MARCH-8. Co-immunoprecipitation (co-IP) assay was performed to test the whether the interaction between MARCH-8 and DR4 was altered in different NDRG1 status. As shown, significant minor DR4 protein was recruited to MARCH-8 in NDRG1 cells than in vector control cells (Figure 5E) while knockdown of NDRG1 led to the opposite results (Figure 5F). To further explore the mechanisms of NDRG1 modulating MARCH-8, we quantified the mRNA levels of MARCH-8 by RT-PCR and protein levels by western blot assays in NDRG1 and shNDRG1 cells. However, no significant difference was detected in either way (Supplementary Figure 1C and 1D). Co-IP was finally performed to test potential interaction between the two molecules. As shown, NDRG1 interacted with MARCH-8 in HCT116 and RKO cells (Figure 5G). Furthermore, immunofluorescence staining consistently showed co-localization (yellow signal) of NDRG1 with MARCH-8 (Figure 5H). These results suggested that NDRG1 suppressed the ubiquitination of DR4 by competitive binding with MARCH-8. Collectively, the data depicted here suggested the accumulation of DR4 protein in NDRG1 cells was attributed to the suppression of its ubiquitination and subsequent degradation.

### NDRG1 sensitized CRC cells to TRAIL

**Figure 6:**
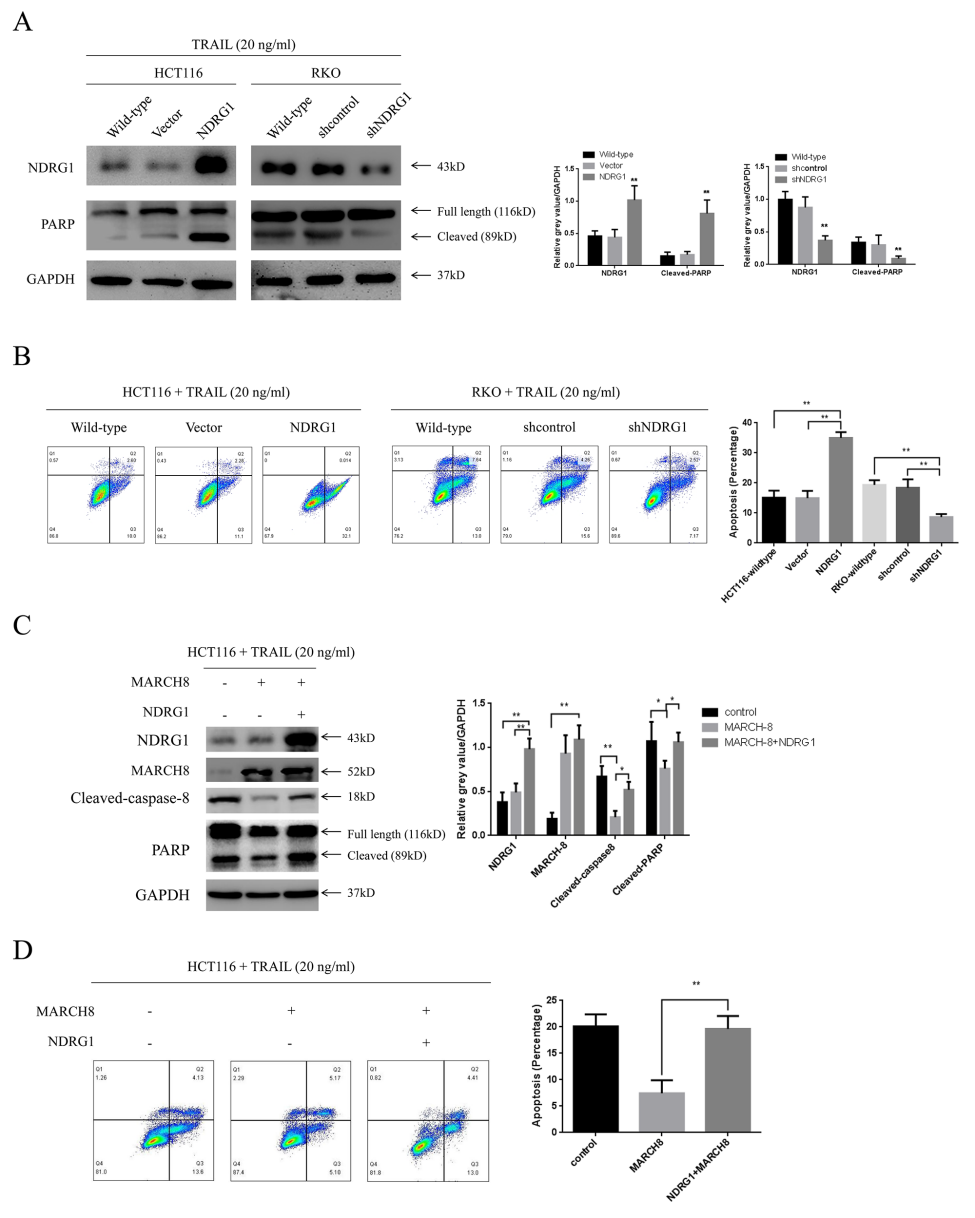
NDRG1 sensitized CRC cells to TRAIL **(A)** Effect of NDRG1 over-expression or knockdown on the response of CRC cells to TRAIL. Apoptosis was analyzed by levels of cleaved-PARP or flow cytometry assay **(B)**. All cells were cultured in the medium containing 20ng/mL TRAIL. HCT116 and RKO wild-type cells were used as blank control. **(C)** NDRG1 attenuated MARCH-8-induced drug resistance. Apoptosis was analyzed with western blots showing levels of cleaved-caspase-8 and -PARP and flow cytometry assay showing apoptotic cell population **(D)**. Data was shown as mean ± S.D. of triplicate experiments. *, *P* < 0.05, **, *P* < 0.01.

The above studies showed that NDRG1 could enhance cell death signal via preserving death receptor 4. Inspired by this, we subsequently presumed that NDRG1 increased sensitivity of CRC cells to reagents targeting death receptor pathway such as TRAIL. To validate this hypothesis, the effect of NDRG1 on the response of CRC cells to TRAIL was tested. Treatment of TRAIL (20 ng/ml) to cell lines followed by western blot detected enhanced cleavage of PARP (Figure 6A), which was a hallmark of apoptosis, in NDRG1 cells. Moreover, larger population of apoptotic cells was observed in NDRG1 over-expressed cells relative to control cells (Figure 6B). In the mean time, NDRG1 knockdown resulted in impaired response of RKO cells to drug treatment (Figure 6A and 6B). More importantly, NDRG1 could attenuate the resistance of MARCH-8 over-expressed cells to TRAIL induced apoptosis (Figure 6C and 6D), providing further evidence that NDRG1 promoted TRAIL-induced apoptosis by targeting MARCH-8.

### NDRG1 increased sensitivity of CRC cells to TRAIL *in vivo*

**Figure 7:**
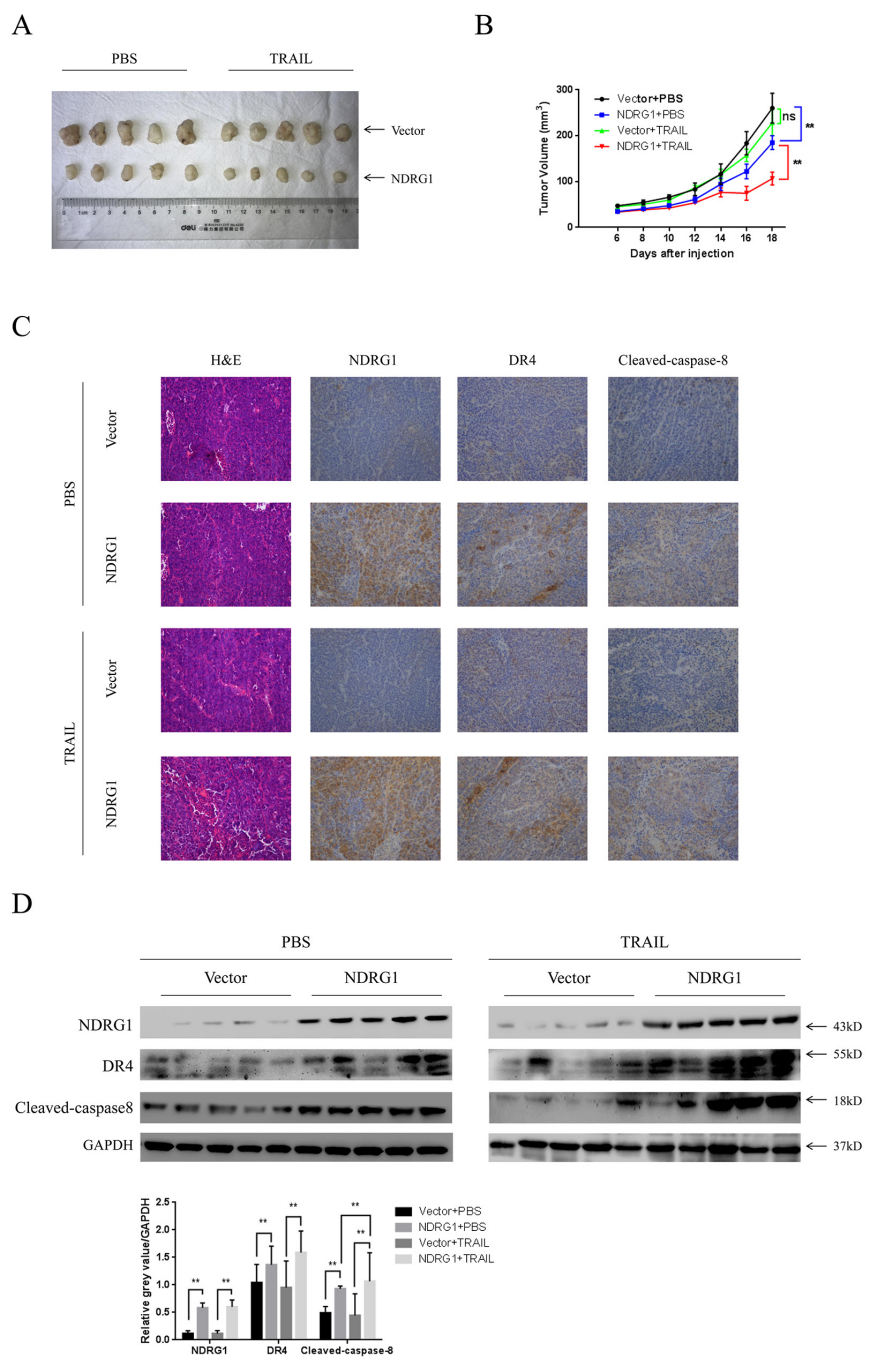
NDRG1 increased sensitivity of CRC cells to TRAIL *in vivo* **(A)** Photograph of xenografts from nude mice that subcutaneously injected with HCT116-Vector and -NDRG1 cells. **(B)** Growth curves of xenograft tumors in nude mice. Mice bearing pre-established tumors (n = 5 per group) were dosed daily for 12 days with intraperitoneal injections of TRAIL (5 mg/kg). During the treatment, tumor volumes were estimated using calipers. Statistical significance was assessed by ANOVA. **(C)** Represent photograph of IHC staining showing NDRG1, DR4 and cleaved-caspase-8 in xenograft tumors from HCT116-Vector or -NDRG1 group. **(D)** Levels of NDRG1, DR4 and cleaved-caspase-8 of xenografts were confirmed by western blot assay.

To validate the effect of NDRG1 on CRC cells *in vivo*, we established a mouse xenograft model. HCT116-Vector and -NDRG1 cells were injected subcutaneously in the flanks of nude mice (Figure 7A). After formation of xenograft tumor (6 days), mice were daily treated with intraperitoneal injection of recombinant human TRAIL (5 mg/kg) or PBS. As shown, xenografts formed by HCT116-NDRG1 cells grew significantly (*P* < 0.01, Figure 7B) slower than those originated from HCT116-Vector cells. Moreover, the treatment of TRAIL significantly suppressed the growth of HCT116-NDRG1 tumors (*P* < 0.01) but not the HCT116-Vector tumors (*P =* 0.087, Figure 7B). Then, the expression levels of NDRG1, DR4 and cleaved-caspase-8 in xenografts were detected by IHC and western blots (Figure 7C and 7D). Caspase-8 cleavage was significantly elevated in HCT116-NDRG1 xenografts, as a consequence of increased DR4 levels in the same cases (Figure 7C and 7D). Collectively, these results suggested NDRG1 could increase the sensitivity of CRC cells to TRAIL *in vivo* and were consistent with our *in vitro* observations.

## DISCUSSION

One of the most crucial advances in cancer research is the recognition that apoptosis determines chemotherapy treatment response. Dysregulation or blockade of apoptosis in cancer cells represents a potential mechanism of resistance to anticancer therapies [22–24]. Thus, our findings in this study that NDRG1 is sufficient to promote TRAIL-induced apoptosis in CRC via up-regulating DR4 are of great importance.

NDRG1 is a potent tumor suppressor gene in multiple types of cancer including CRC. The antitumor activity of this protein involves its suppressive effects on several tumorigenic signaling pathways [25–28]. Our first goal was to evaluate the clinical significance of NDRG1 in CRC. Initially, our data showed that NDRG1 was inversely correlated to tumor sizes, local invasion and TNM stage. Generally, decrease in tumor sizes may be caused by perturbations of the cell growth or induction of cell death. Moreover, it has been reported that NDRG1 was necessary to P53-induced apoptosis, suggesting that NDRG1 might suppress cell growth via triggering apoptosis in cancer cells. As expected, dramatic apoptotic cell population was detected in NDRG1-overexpressed cells by various methods.

Pathscan array is currently considered as a powerful tool to identify potential cancer biomarkers and prognostic targets [29]. We undertook this systemic approach to discover the potential protein alterations in CRC cells over-expressing NDRG1. As elevated levels of cleaved-caspase-7 were revealed, we hypothesized NDRG1 might enhance apoptosis via triggering the death receptor pathway [14]. As expected, the protein level of DR4 was up-regulated in NDRG1 cells. Death receptor 4 is one of the tumor necrosis factor-related apoptosis-inducing ligand (TRAIL) receptors and triggers apoptosis upon the extracellular apoptotic stimulations [30–32]. The ligation of natural ligands with death receptors lead to the recruitment of the adaptor protein Fas-associated death domain (FADD), which recruits caspase-8 zymogens to form death-inducing signaling complexes (DISCs) [33]. Caspase-8 can be cleaved and activated at DISCs and subsequently activate downstream effector caspases [34]. In turn, caspases activation resulted in PARP cleavage, nuclear condensation, and eventually, the induction of apoptosis.

We next questioned the mechanisms of NDRG1 up-regulating DR4. NDRG1-depleted CRC cells were treated with MG132, a classical proteasomal inhibitor [35] or BafA (lysosomal inhibitor [36]). As reported above, MG132 rescued the decreased DR4 protein level caused by NDRG1 depletion whereas BafA had no effect on DR4 degradation, which meant DR4 degraded in a proteasome-dependent manner in NDRG1-knockdown cells. Accordingly, we found that NDRG1 suppressed DR4 ubiquitination and subsequent proteasomal degradation. The membrane-associated RING-CH (MARCH) proteins belong to a recently discovered family of RING-finger ubiquitin ligases [37, 38]. Several members of this novel E3 ubiquitin ligase family have been shown to ubiquitinate and down-regulate transmembrane proteins, such as CD86, CD166, and death receptors [39]. As previous studies reported, MARCH-8 directly interacted with DR4 and mediated its poly-ubiquitination and degradation [21]. Therefore, we hypothesized that NDRG1 might regulate the protein levels of DR4 via modulating MARCH-8. This idea was supported by our observation in the current study that NDRG1 could suppress DR4 ubiquitination via directly interacting with MARCH-8. Thus, CRC cells may be more sensitive to apoptosis initiated by death receptors and its ligands such as TRAIL.

TRAIL is a member of the tumor necrosis factor (TNF) superfamily of cytokines which induces apoptosis upon binding to its death domain-containing transmembrane receptors such as death receptors 4 and 5 (DR4, DR5)[40]. Importantly, TRAIL preferentially induces apoptosis in cancer cells while exhibiting little toxicity in normal cells [41]. Possessing this unique ability, TRAIL seems to be a very promising candidate to be included into chemotherapy regimens of multiple types of cancer. However, previous studies have shown that many cancer types are resistant to the apoptotic effects of TRAIL [42, 43]. Thus, it is important to investigate the mechanisms involved in the resistance and develop agents that sensitize cancer cells to TRAIL-induced apoptosis. As we reported, NDRG1 could up-regulate DR4 and sensitize CRC cells to TRAIL *in vitro* and *in vivo*. Therefore, NDRG1 is sufficient to overcome the threshold to modulate TRAIL-induced apoptosis in colorectal cancer.

In conclusion, our study highlighted the pivotal role of NDRG1 in activating death receptor apoptotic pathway. Thus, NDRG1 could serve as a response predictor of CRC cells to TRAIL. In addition, we believed that TRAIL was a very promising candidate to be included into chemotherapy regimens combating CRC, especially in NDRG1-positive patients. Nevertheless, this conclusion needs to be confirmed in further clinical trial. We are actively pursuing these findings in greater depth.

## MATERIALS AND METHODS

### Tissue samples and immunohistochemistry (IHC)

A total of 116 CRC patients were enrolled. Tumor tissues and adjacent normal tissues (>5 cm away from carcinomas) were collected from patients undergoing radical surgery at Shanghai East Hospital and Shanghai Ruijin Hospital. Portions of specimens were fixed in the formalin for the analysis of immunohistochemistry. Immunohistochemistry (IHC) staining was performed using formalin-fixed, parafin-embedded tissue sections. In chief, tissue sections were deparaffinized, rehydrated, and microwaved-heated in sodium citrate buffer (10mmol/L, pH6.0) for antigen retrieval. Then, the slides were incubated with primary antibody. Counterstaining was performed using hematoxylin eosin stain. The anti-NDRG1 antibody (1:300) was from Cell Signaling Technology (Beverly, MA, USA). This study was approved by the Ethical Committee of Ruijin Hospital Affiliated to Shanghai Jiao Tong University School of Medicine. Written consent for experimental procedures was obtained from all enrolled patients.

Two independent pathologists who were blinded from any data of this study examined the cellular location of NDRG1 and compared the staining between the CRC tissue and normal tissue in each case. IHC scoring was based on the proportion of staining (0 = 0%, 1 ≤ 25%, 2 = 25% to 50%, 3 = 51% to 75%, 4 ≥ 75% positive cells) and the staining intensity (0 = no staining, 1 = weak, 2 = moderate, 3 = strong). The scores for staining intensity and percentage were multiplied. An overall score of ≤ 6 was defined as negative, while a score > 6 was defined as positive.

### Cell lines

CRC cell lines (SW480, SW620, HCT116, SW1116, HT29, and RKO) were purchased from ATCC (Manassas, VA, USA) and preserved in Shanghai institute of digestive surgery. SW480 and SW1116 cells were cultured in RPMI-1640 medium. HT29 and HCT116 were cultured in McCoy’s 5A medium, RKO cells were cultured in Dulbecco’s modified Eagle’s medium (DMEM). SW620 cells were cultured in Leibovitz’s L-15 medium. All medium were purchased from Invitrogen (Carlsbad, CA, USA) and supplemented with 10 % fetal bovine serum (FBS, HyClone, Logan, UT, USA), 100 U/ml penicillin and 100 μg/ml streptomycin. Cells were maintained in a 37°C incubator with 5% CO_2_.

### Plasmids and transfection

The full length NDRG1 cDNA was synthesized by RT-PCR from normal colon epithelial cells (NCM460). Then the cDNA was sub-cloned into the pcDNA3.1 plasmid (Invitrogen, Carlsbad, CA, USA). The shRNA sequences were synthesized and then cloned into pLKO.1 plasmid (Sigma Aldrich, St. Louis, MO, USA). The NDRG1/shNDRG1 plasmids and corresponding empty vector were transfected into CRC cells (HCT116 and RKO) using Lipofectamine 2000 reagent (Invitrogen) following the manufacturer’s protocol. Stable clones were selected by puromycin (1 μg/ml). All these stable transfects were tested regularly by western analysis to ensure the efficiency of over-expression or knockdown.

### Pathscan array

Conducting of pathscan array was strictly followed the manufacturer’s protocol. In brief, the glass slides containing detecting antibody matrix was prepared and assembled. Then the blocking buffer was added in each well and incubated in room temperature for 15 minutes. After washing, a total protein of 30μg was added in each well and incubated at 4°C overnight on an orbital shaker. Then the slide was washed with PBS and placed on a plastic dish. The fluorescence intensity was captured using an Oddessy fluorescent imaging system (LI-COR Biotechnology, Lincoln, NE, USA).

### Quantitative real-time PCR (qRT-PCR)

Total RNA was extracted using TRIzol reagent and cDNA synthesis was performed using a reverse transcription kit (Promega, Madison, WI, USA) according to the manufacturer’s instructions. The mRNA level of NDRG1 was measured using the SYBR Green PCR Master Mix (Applied Biosystems, Waltham, MA, USA) and the Applied Biosystems 7900HT sequence detection system (Applied Biosystems). Relative mRNA level was evaluated using the 2^−ΔΔCt^ method and normalized to glyceraldehyde 3-phosphate dehydrogenase (GAPDH). The primers used in our study were listed in Supplementary Table 1.

### Apoptosis analysis

CRC cells were trypsinized and washed with serum-containing medium, and then centrifuged for 5 min at 1500rpm. Next, the cells were resuspended in Binding Buffer and stained for 15 min at room temperature using the Annexin V-FITC/PI Apoptosis Kit (BD, USA) according to the protocols. The number of apoptotic cells was detected using BD FACS Vantage System. Both PI- and annexin V-negative cells (quadrant 4) were intact cells, PI-negative and annexin V-positive cells were considered as early apoptotic (quadrant 3) cells, both PI- and annexin V-positive (quadrant 2) cells were late apoptotic cells, PI-positive and annexin V-negative cells were considered to be mechanically injured (quadrant 1) during the experiment.

Moreover, the apoptotic cells were also detected by the TUNEL assay using *In situ* Cell Death Detection Kit (Roche Applied Science, USA), according to the manufacturer’s protocol. Nuclei were detected using DAPI staining.

### Xenograft experiments

Mice were maintained at the animal experiment center of Ruijin Hospital under standard conditions following institutional guidelines. A total of 1×10^7^ HCT116-NDRG1 cells or HCT116-Vector cells were injected subcutaneously into the flanks of nude mice (male BALB/c nu/nu nude mice, 4-week-old). After we confirmed the success of xenograft (6 days after injection), mice were treated daily for 14 days with intraperitoneal injections of 5 mg/kg of TRAIL dissolved in PBS. Another group of mice were treated with 10μl PBS and served as a blank control. Tumor size was measured every other day using calipers. Tumor volume (V) was determined by measuring the length and width of the tumor and using the formula $V=\left({\text{width}}^{2}\times \text{length}\right)/2$. All the mice were sacrificed 16 days after treatment and tumor grafts were excised for further research. Antibodies used for IHC staining of xenografts were anti-NDRG1 (WH0010397M3, Sigma Aldrich, St. Louis, MO, USA), anti-death receptor 4 (#42533, Cell Signaling Technology), anti-cleaved-caspase-8 (#9930, Cell Signaling Technology). Recombinant human TRAIL was purchased from Thermo Fisher Scientific (PHC1634).

### Western blot assay

For routine western blot, cells were lysed in cell lysis solution (Thermo Scientific, MA, USA). Total protein (50μg) was resolved by 12.5 % SDS-PAGE and transferred onto 0.22-lm polyvinylidene fluoride membranes (Millipore, MA, USA). The membranes were probed with appropriate antibodies overnight at 4 °C. After washing for three times, the membrane was incubated in HRP-labeled secondary antibody for 2 hours at room temperature. Protein level was normalized to GAPDH. Antibodies used in our study were listed as follows: Apoptosis Antibody Sampler Kit (#9930) including anti-PARP, anti-cleaved-caspase-3, and anti-cleaved-caspase-8 antibodies were purchased from Cell Signaling Technology. Anti-Bcl-2 (#15071), anti-Puma (#12450), anti-Bax (#5023), anti-DR4 (#42533), anti-DR5 (#8074), antibodies were also obtained from Cell Signaling Technology. Anti-NDRG1 antibody (WH0010397M3) was from Sigma Aldrich (St. Louis, MO, USA).

### Immunoprecipitation

Immunoprecipitation was performed by an established method. In chief, cells were washed with ice-cold PBS and lysed using NP40-containing lysis buffer with protease inhibitors. Lysates (300μg) were incubated with monoclonal DR4 antibody (Cell Signaling Technology) overnight at 4°C. This mixture was added to 30μL of protein G Beads (Santa Cruz) and incubated for 4h/4°C. The Beads were then washed three times with ice-cold PBS, resuspended in SDS-loading buffer and incubated at 100°C/5min. The supernatant was separated on a 12.5% Bris-Tris gel. Ubiquitin-conjugates were revealed by western blotting using anti-unbiquintin (ab7780, abcom, Cambrige, USA).

For co-immunoprecipitation assay (co-IP), cells were harvested with immunoprecipitation lysis buffer (20 mM Tris-HCl, pH 7.6; 150 mM NaCl; 1 mM EDTA; 0.5% NP-40; 10% glycerol; 1 mM PMSF; protease inhibitor cocktail). After sonication, the lysates were centrifuged (17,000g, 15min) at 4°C. Equal amounts of cell lysates were incubated for 12h with corresponding primary antibodies or IgG. Subsequently, protein A/G agrose (Santa Cruz) was added and incubated with cell lysates for 4h. All incubations were performed at 4°C with gentle rotation. After washing 3 times with RIPA buffer, proteins were eluted and subjected to western bloting.

### Immunofluorescence (IF)

Cells were fixed with 4% paraformaldehyde and permeabilized using 0.5% Triton X-100. After blocked with 5% BSA, the cells were incubated with the primary antibody, followed by the Alexa Fluor–conjugated IgG secondary antibody. Images were observed by fluorescence microscopy. Anti-MARCH-8 antibody was from Abcam (ab99229). The intensity of fluorescence was analyzed with ImageJ software.

### Statistical analysis

IBM SPSS Statistical software (version 19.0) was utilized for statistical analysis. Protein and mRNA levels were compared using two-tailed Student *t* test. Correlations between NDRG1 expression in CRC tissues and clinical-pathological parameters were analyzed using the Pearson Chi-square (χ2) test. Overall survival was assessed by Kaplan-Meier method, and difference between survival curves was determined by using the log-rank test. Differences with a *P* value < 0.05 were considered to be statistically significant.

## SUPPLEMENTARY MATERIALS FIGURE AND TABLE



## Abbreviations

CRC: colorectal cancer
NDRG1: N-myc downstream regulated gene-1
RT-PCR: reverse transcription-polymerase chain reaction
TRAIL: tumor necrosis factor-related apoptosis-inducing ligands
IHC: immunohistochemistry
