# Supplementary material for: N-myc downstream-regulated gene 1 promotes apoptosis in colorectal cancer via up-regulating death receptor 4
Source: Oncotarget. 2017 Jul 28;8(47):82593–608. doi: 10.18632/oncotarget.19658 (PMC5669913; doi:10.18632/oncotarget.19658)
Supplement: Supplementary file 1 [file oncotarget-08-82593-s001.pdf]

N-myc downstream-regulated gene 1 promotes apoptosis in colorectal cancer via up-regulating death receptor 4

SUPPLEMENTARY MATERIALS

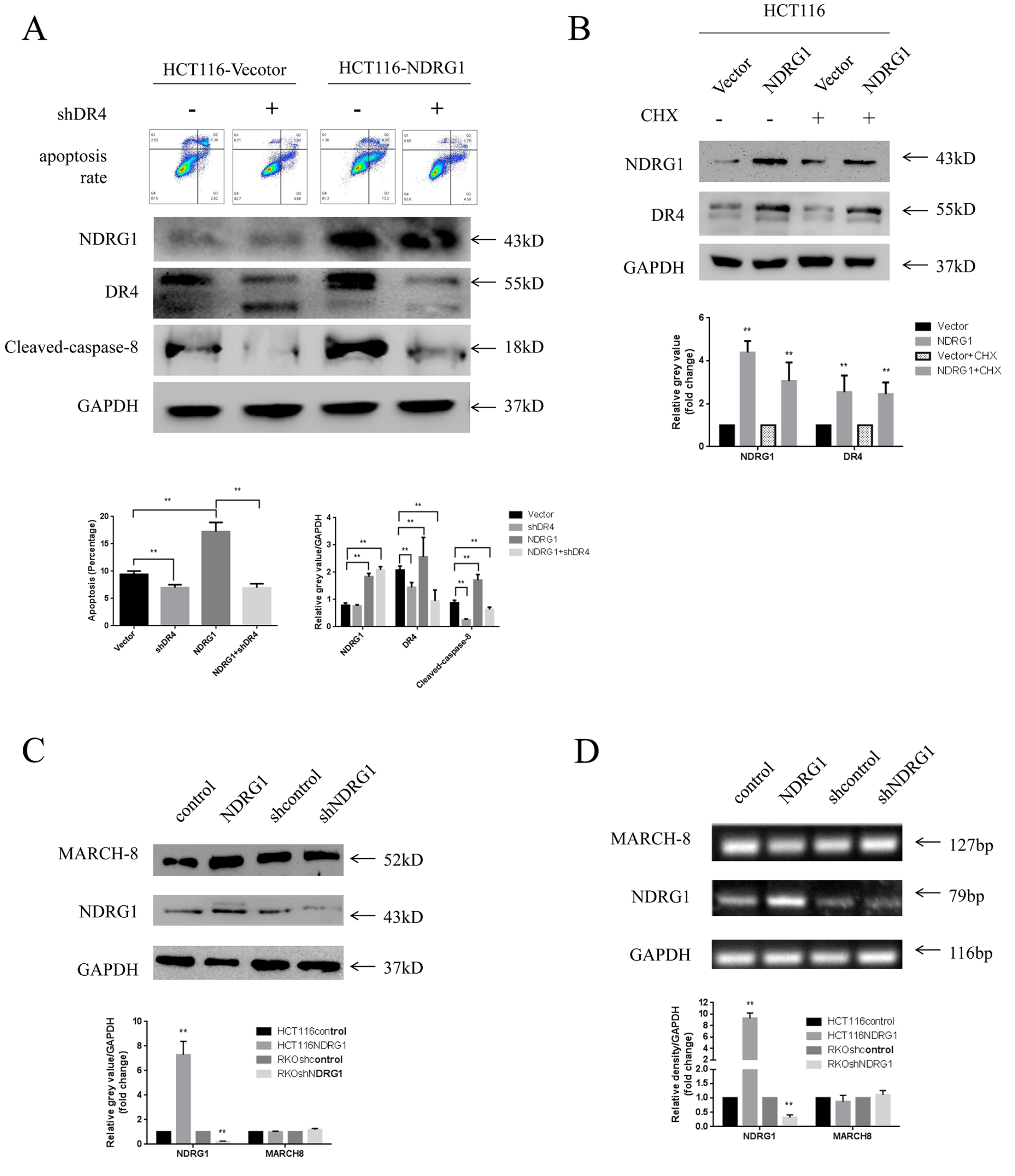

Supplementary Figure 1: (A) Knockdown of DR4 decreased the apoptosis in HCT116-vector and -NDRG1 cells. Apoptosis was measured by western blots showing levels of cleaved-caspase-8 and flow cytometry assay showing apoptotic cell population. **(B)** Western blots showing DR4 protein levels in HCT116-vectro and –NDRG1 cells with or without CHX treatment. **(C)** Effect of NDRG1 on the protein levels of MRACH-8 in CRC cells. **(D)** mRNA level of MARCH-8 in NDRG1 over-expressed and knockdown cells. Error bars represent the mean ± S.D. of triplicate experiments.

Supplementary Table 1: Primers used for PCR

Gene name	Forward	Reverse
NDRG1	CTCCTGCAAGAGTTTGATGTCC	TCATGCCGATGTCATGGTAGG
Bcl-2	CCAGCGTATATCGGAATGTGG	CCATGTGATACCTGCTGAGAAG
Bax	CCCGAGAGGTCTTTTTCCGAG	CCAGCCCATGATGGTTCTGAT
Mcl-1	TGCTTCGGAAACTGGACATCA	TAGCCACAAAGGCACCAAAAG
Puma	GACCTCAACGCACAGTACGAG	AGGAGTCCCATGATGAGATTGT
DR4	TCCTGAGCTGCATTGCACTC	CTCTTCGCAGTTCCTGGATTAAA
DR5	ACTCGTGTCATCAGCGACTTG	GCAACAGTAGGTTTCCTTGTGT
c-FLIP	GACAGAGCTTCTTCGAGACAC	GCTCGGGCATACAGGCAAAT
GAPDH	GGAGCGAGATCCCTCCAAAAT	GGCTGTTGTCATACTTCTCATGG
